# Sex-specific association between atherogenic index of plasma and risk of newly diagnosed abdominal aortic aneurysm: a large population-based cohort study

**DOI:** 10.1186/s40001-025-02586-4

**Published:** 2025-05-05

**Authors:** Peng Qiu, Hongbin Guo, Chao Zhu, Yijun Liu, Jiazhen Zheng, Hongji Pu, Xinwu Lu, Qun Huang, Guang Liu, Kaichuang Ye, Zhen Zhou

**Affiliations:** 1 Department of Vascular Surgery, Shanghai Ninth People’s Hospital, Shanghai JiaoTong University School of Medicine, 639 Zhizaoju Rd, Shanghai, 200011 China; 2 Department of Neuroscience, School of Translational Medicine, Monash University, 99 Commercial Rd, Melbourne, VIC 3004 Australia; 3 Bioscience and Biomedical Engineering Thrust, Systems Hub, The Hong Kong University of Science and Technology (Guangzhou), No. 1 Du Xue Road, Nansha District, Guangzhou, Guangdong China; 4 School of Public Health and Preventive Medicine, Monash University, 553 St Kilda Rd, Melbourne, VIC 3004 Australia

**Keywords:** Atherogenic index of plasma, Abdominal aortic aneurysm, Atherosclerosis, Sex, Risk factor

## Abstract

**Objectives:**

Atherosclerosis of aortic wall has been suggested as a key pathological feature of abdominal aortic aneurysm (AAA). We conducted a first-ever prospective cohort study aiming at assessing the sex-specific association between atherogenic index of plasma (AIP) and risk of newly diagnosed AAA.

**Methods:**

This study included 193,013 male and 226,785 female participants from the UK Biobank. AIP was calculated as a ratio of logarithmically transformed triglycerides to high-density lipoprotein-cholesterol. The outcome of interest was new AAA, identified by ICD-10 and OPCS-4 code, or by AAA-related death. All analyses were sex-stratified: Multivariable Cox proportional-hazard models were employed to assess the association between baseline AIP and AAA risk. Harrell’s c index was estimated to assess the value of AIP added to the discrimination of AAA prediction model.

**Results:**

Over an average follow-up of 15.3 years, 1931 (1.00%) new AAA cases were recorded in males and 424 (0.19%) in females. In the fully adjusted models, compared with the bottom AIP quintile, HRs (95% CI) of newly diagnosed AAA was 1.67 (1.41, 1.96) in males and 1.75 (1.22, 2.52) in females within the top quintile. Subgroup analysis found smoking status significantly modified the association in females, with association existing only in female ever-smokers. Adding AIP into prediction model comprising age, smoking, and CVD history significantly improved the discrimination in males and male high-risk subgroups and in female ever-smokers (*p* < 0.05).

**Conclusions:**

This study highlights the potential of AIP as a biomarker for AAA and its utility in identifying high-risk individuals qualified for AAA screening.

**Supplementary Information:**

The online version contains supplementary material available at 10.1186/s40001-025-02586-4.

## Introduction

Ruptured abdominal aortic aneurysm is health emergency with death rate reaching up to 80%. AAA is asymptomatic before rupture indicating screening of high population as a fundamental for prevention of this life-threatening diseases. Despite the complexity of pathogenesis, AAA has been suggested to be invariably associated with atherosclerosis of aortic wall, which can induce adaptive remodelling of the arterial extracellular matrix, resulting in arterial expansion to maintain normal lumen diameter and shear stress levels [1, 2]. Atherogenic index of plasma, calculated as a ratio of logarithmically transformed triglycerides to high-density lipoprotein (HDL)-cholesterol, is a surrogate marker of the concentration of small dense low-density lipoprotein (LDL) and a novel risk factor of atherosclerosis and metabolic syndrome [3, 4]. AIP has garnered increased interests recently due to its predictive value of cardiovascular disease (CVD) independent of other CVD risk factors and its superiority to the standard atherosclerotic lipid indices [5–8]. However, there is no study that has ever investigated the ability of AIP for predicting AAA. Given the rarity of AAA, large-scale studies with extensive follow-up are essential to establish a robust evidence base for this critical clinical question. Leveraging data from UK biobank, we investigated the association between baseline AIP levels and newly identified AAAs. Subgroup analyses were conducted by sex, diabetes and other AAA risk factors.

## Methods

### Data source

UK Biobank is a large-scale, population-based, nationwide. Prospective cohort study that recruited approximately half a million participants across England, Scotland, and Wales between 2007 and 2010, who were registered with the National Health Service (NHS) general practitioners during the recruitment period [9]. Participants were aged between 37 and 73 years at the time of recruitment and the follow-up remains ongoing. UK Biobank received ethical approval from the Northwest Multi-Centre Research Ethics Committee (11/NW/03820). All participants gave written informed consent before enrolment in the study, which was conducted in accordance with the principles of the Declaration of Helsinki.

Participants’ characteristics were collected at the assessment centre when they were recruited, through a self-administered, touch-screen questionnaire and face-to-face interview to collect information on their lifestyle, health, medical and socioeconomic characteristics. Trained research staff measured their height, weight, and blood pressure and obtained samples of blood, saliva and urine.

This study included all UK Biobank participants who did not have a history of AAA at baseline and had available data on HDL-c and triglycerides to generate AIP and covariates used for adjustment in outcome models.

### Study exposure

The study exposure was atherogenic index of plasma (AIP), calculated as logarithmically transformed triglycerides divided by HDL-c. Higher AIP levels reflect a greater risk of atherosclerosis. In UK Biobank, serum lipid profiles were measured using non-fasting blood samples, which were collected from participants at *baseline* recruitment according to validated standardised procedures. There was no follow-up measurement of HDL-c and other blood serum biomarkers. Details on serum sample handling and assays were described elsewhere [10].

### Outcomes

The outcome of interest was newly diagnosed AAA. AAA cases were identified using the International Classification of Disease (ICD)-10 codes for ruptured (I71.3) and non-ruptured AAA (I71.4) for AAA, the Office of Population, Censuses and Surveys (OPCS)-4 Classification of Interventions and Procedures codes related to AAA (L18, L19, L254, L27, L28, L464), or by identifying deaths attributed to AAA [11]. An AAA-related death was defined as a death, where the primary cause was identified as an AAA. Dates and causes of death were obtained from death certificates held by the NHS Information Centre for participants from England and Wales and the NHS Central Register Scotland for participants from Scotland [12]. Participants were followed from the date of participants accessing assessment centre until the new diagnosis of AAA, death, or the time of data censoring (1 st June 2024), whichever came first.

### Baseline covariates

Baseline covariates include age, race, smoking status (never, former, current), alcohol consumption (never, former, current), education level (university or college degree, below university or college degree), Townsend deprivation index (lower than median, median or higher), hypertension (defined as systolic/diastolic blood pressure [SBP/DBP] ≥ 140/90 or self-reported use of antihypertensive medications), diabetes (defined as HbA1c ≥ 48 mmol/L, self-reported diabetes or use of insulin), CVD history ascertained by ICD-10 codes (defined as self-reported historic angina, myocardial infarction, stroke, or transient ischemic attack), body mass index (BMI) categories (< 25 kg/m^2^ underweight or normal, 25 to < 30 kg/m^2^ overweight, ≥ 30 kg/m^2^ obese), self-reported use of lipid-lowering medication, C-reactive protein (CRP) levels.

### Statistical analysis

All analyses were conducted in males and females separately. Baseline characteristics of study participants were presented across quintiles of AIP. For continuous variables, data were presented as mean (standard deviation, SD) if normally distributed and median (interquartile, IQR) if skewed. Discrete variables were reported as count and percentage.

Stepwise Cox proportional-hazards models were used to investigate the sex-stratified association between AIP and newly diagnosed AAA: (1) Model 1: unadjusted; (2) Model 2: adjusting for all nonlipid covariates (3) Model 3: adjusting for all nonlipid covariates and directly measured LDL-c. AIP was either analysed as a continuous (per 1 SD increase) or categorical variable (by quintile). Hazard ratios (HRs) with 95% confidence intervals (CIs) were reported. Nelson–Aalen cumulative hazard curves for AAA by quintiles of AIP were plotted. Proportional-hazards assumption was checked by scaled Schoenfeld residuals, and no violation of assumption was observed.

Restricted cubic spline was drawn to visualise any potential non-linear relationship between AIP and newly diagnosed AAA in both sexes. Three knots were placed at 10 th, 50 th and 90 th percentiles of the AIP distribution according to the Harrell’s principle [13]. The number of knots was determined according to the Akaike Information Criterion and Bayesian Information Criterion values, with lower values indicating better model fit.

Sex-stratified subgroup analyses were conducted by vascular risk factors including age, smoking, obesity, diabetes, hypertension, CVD history, lipid-lowering medication use and CRP, to determine whether the AIP–AAA association was modified by these risk factors. *p* values for interaction was calculated by adding a productive term of AIP × stratifying factor into the fully adjusted Cox model.

The predictive value of AIP added onto typical AAA risk factors including smoking, age and CVD history [14] was assessed by comparing the discriminatory ability measured by Harrell’s C statistics between the Cox models including the typical AAA risk factors with and without adding AIP. The category-free net reclassification index (cf-NRI) and integrated discrimination improvement (IDI) were additionally computed to assess the enhanced discriminatory performance of the new model that included AIP.

All statistical tests were two-sided, and a *p* < 0.05 was considered statistically significant. Analyses were performed using STATA/SE 18.0 for windows (StataCorp, College Station, TX: StataCorp LLC).

## Results

### Baseline characteristics

Of 501,801 participants with no history of AAA, 419,798 with complete baseline data on AIP and covariates were included in this study. This included 193,013 male participants and 226,785 female participants. In both male and female participants, those with higher AIP levels were more likely to be current smokers, but less likely to be current drinkers, statin users, and less educated. They were also more likely to have hypertension, diabetes, obesity, and CVD history, and have higher CRP levels. Female participants with higher AIP levels also appeared to be older but this trend was not observed in male participants (Table 1).

**Table 1:** Baseline characteristics in male participants, stratified by sex and quintiles of atherogenic index of plasma (AIP)

	Males						Females					
	Total	Quintile 1	Quintile 2	Quintile 3	Quintile 4	Quintile 5	Total	Quintile 1	Quintile 2	Quintile 3	Quintile 4	Quintile 5
No. of participants	193,013	38,604	38,602	38,602	38,603	38,602	226,785	45,357	45,357	45,358	45,356	45,357
AIP, mean [21]	0.32 (0.67)	− 0.61 (0.28)	− 0.05 (0.12)	0.31 (0.10)	0.68 (0.11)	1.26 (0.31)	− 0.12 (0.63)	− 0.96 (0.22)	− 0.49 (0.10)	− 0.16 (0.10)	0.20 (0.12)	0.79 (0.32)
AIP, range	(− 2.30 to 3.22)	(− 2.30 to − 0.26)	(− 0.26 to 0.14)	(0.14 to 0.49)	(0.49 to 0.89)	(0.89 to 3.22)	(− 2.43 to 3.22)	(− 2.44 to − 0.67)	(− 0.67 to − 0.32)	(− 0.32 to 0.01)	(0.01 to 0.41)	(0.41 to 2.81)
HDL cholesterol, mean [21], mmol/L	1.28 (0.31)	1.63 (0.32)	1.37 (0.23)	1.25 (0.21)	1.15 (0.19)	1.01 (0.18)	1.59 (0.38)	1.99 (0.36)	1.73 (0.28)	1.58 (0.26)	1.44 (0.24)	1.24 (0.22)
Triglycerides, median (IQR), mmol/L	1.69 (1.18–2.44)	0.88 (0.74–1.03)	1.29 (1.13–1.46)	1.69 (1.50–1.90)	2.23 (1.97–2.52)	3.39 (2.87–4.16)	1.33 (0.96–1.89)	0.76 (0.65–0.88)	1.04 (0.92–1.17)	1.33 (1.18–1.50)	1.73 (1.53–1.96)	2.60 (2.20–3.17)
Age, mean [21]	56.7 (8.2)	56.7 (8.3)	57.0 (8.2)	57.0 (8.2)	56.8 (8.1)	55.9 (8.1)	56.4 (8.0)	54.3 (8.1)	55.6 (8.1)	56.6 (8.0)	57.5 (7.8)	57.9 (7.5)
Race, *n* (%)												
White	182,166 (94.4%)	36,246 (93.9%)	36,436 (94.4%)	36,522 (94.6%)	36,540 (94.7%)	36,422 (94.4%)	214,444 (94.6%)	42,639 (94.0%)	42,940 (94.7%)	42,914 (94.6%)	43,020 (94.8%)	42,931 (94.7%)
Black	2807 (1.5%)	1101 (2.9%)	699 (1.8%)	463 (1.2%)	315 (0.8%)	229 (0.6%)	3682 (1.6%)	1264 (2.8%)	909 (2.0%)	704 (1.6%)	506 (1.1%)	299 (0.7%)
Asian	4711 (2.4%)	622 (1.6%)	807 (2.1%)	963 (2.5%)	1087 (2.8%)	1232 (3.2%)	4456 (2.0%)	559 (1.2%)	691 (1.5%)	869 (1.9%)	999 (2.2%)	1338 (2.9%)
Mixed	928 (0.5%)	207 (0.5%)	195 (0.5%)	150 (0.4%)	185 (0.5%)	191 (0.5%)	1515 (0.7%)	383 (0.8%)	290 (0.6%)	306 (0.7%)	285 (0.6%)	251 (0.6%)
Others	2401 (1.2%)	428 (1.1%)	465 (1.2%)	504 (1.3%)	476 (1.2%)	528 (1.4%)	2688 (1.2%)	512 (1.1%)	527 (1.2%)	565 (1.2%)	546 (1.2%)	538 (1.2%)
Smoking, *n* (%)												
Never	95,013 (49.2%)	20,414 (52.9%)	19,760 (51.2%)	18,988 (49.2%)	18,460 (47.8%)	17,391 (45.1%)	135,322 (59.7%)	28,359 (62.5%)	27,935 (61.6%)	27,387 (60.4%)	26,569 (58.6%)	25,072 (55.3%)
Former	74,131 (38.4%)	14,049 (36.4%)	14,523 (37.6%)	15,111 (39.1%)	15,291 (39.6%)	15,157 (39.3%)	71,351 (31.5%)	14,294 (31.5%)	14,115 (31.1%)	14,120 (31.1%)	14,282 (31.5%)	14,540 (32.1%)
Current	23,869 (12.4%)	4141 (10.7%)	4319 (11.2%)	4503 (11.7%)	4852 (12.6%)	6054 (15.7%)	20,112 (8.9%)	2704 (6.0%)	3307 (7.3%)	3851 (8.5%)	4505 (9.9%)	5745 (12.7%)
Alcohol use, *n* (%)												
Never	5284 (2.7%)	809 (2.1%)	931 (2.4%)	1039 (2.7%)	1105 (2.9%)	1400 (3.6%)	13,055 (5.8%)	1555 (3.4%)	2118 (4.7%)	2586 (5.7%)	2986 (6.6%)	3810 (8.4%)
Former	6723 (3.5%)	1027 (2.7%)	1287 (3.3%)	1305 (3.4%)	1461 (3.8%)	1643 (4.3%)	8161 (3.6%)	1064 (2.3%)	1362 (3.0%)	1476 (3.3%)	1845 (4.1%)	2414 (5.3%)
Current	181,006 (93.8%)	36,768 (95.2%)	36,384 (94.3%)	36,258 (93.9%)	36,037 (93.4%)	35,559 (92.1%)	205,569 (90.6%)	42,738 (94.2%)	41,877 (92.3%)	41,296 (91.0%)	40,525 (89.3%)	39,133 (86.3%)
University/college degree, *n* (%)	24,243 (12.6%)	5414 (14.0%)	5115 (13.3%)	4780 (12.4%)	4588 (11.9%)	4346 (11.3%)	23,775 (10.5%)	6091 (13.4%)	5311 (11.7%)	4682 (10.3%)	4116 (9.1%)	3575 (7.9%)
Townsend deprivation index, *n* (%)												
Less deprived (< median)	96,626 (50.1%)	19,249 (49.9%)	19,778 (51.2%)	19,727 (51.1%)	19,508 (50.5%)	18,364 (47.6%)	113,565 (50.1%)	23,654 (52.2%)	23,385 (51.6%)	23,205 (51.2%)	22,350 (49.3%)	20,971 (46.2%)
More deprived (≥ median)	96,387 (49.9%)	19,355 (50.1%)	18,824 (48.8%)	18,875 (48.9%)	19,095 (49.5%)	20,238 (52.4%)	113,220 (49.9%)	21,703 (47.8%)	21,972 (48.4%)	22,153 (48.8%)	23,006 (50.7%)	24,386 (53.8%)
Hypertension, *n* (%)	119,252 (61.8%)	21,177 (54.9%)	22,962 (59.5%)	24,045 (62.3%)	25,221 (65.3%)	25,847 (67.0%)	109,808 (48.4%)	16,572 (36.5%)	19,212 (42.4%)	21,804 (48.1%)	24,510 (54.0%)	27,710 (61.1%)
Diabetes, *n* (%)	14,806 (7.7%)	1680 (4.4%)	2134 (5.5%)	2773 (7.2%)	3394 (8.8%)	4825 (12.5%)	9570 (4.2%)	816 (1.8%)	873 (1.9%)	1320 (2.9%)	2150 (4.7%)	4411 (9.7%)
CVD history, *n* (%)	14,782 (7.7%)	2198 (5.7%)	2731 (7.1%)	3022 (7.8%)	3309 (8.6%)	3522 (9.1%)	6558 (2.9%)	652 (1.4%)	915 (2.0%)	1233 (2.7%)	1549 (3.4%)	2209 (4.9%)
BMI, *n* (%)												
Underweight/Normal	48,507 (25.1%)	18,127 (47.0%)	12,042 (31.2%)	8615 (22.3%)	5966 (15.5%)	3757 (9.7%)	90,059 (39.7%)	29,867 (65.8%)	23,106 (50.9%)	17,475 (38.5%)	12,332 (27.2%)	7279 (16.0%)
Overweight	95,579 (49.5%)	16,651 (43.1%)	19,770 (51.2%)	20,315 (52.6%)	20,059 (52.0%)	18,784 (48.7%)	83,350 (36.8%)	12,376 (27.3%)	16,002 (35.3%)	18,152 (40.0%)	18,693 (41.2%)	18,127 (40.0%)
Obese	48,927 (25.3%)	3826 (9.9%)	6790 (17.6%)	9672 (25.1%)	12,578 (32.6%)	16,061 (41.6%)	53,376 (23.5%)	3114 (6.9%)	6249 (13.8%)	9731 (21.5%)	14,331 (31.6%)	19,951 (44.0%)
Use of LLT, *n* (%)	43,243 (22.4%)	6625 (17.2%)	8072 (20.9%)	8815 (22.8%)	9498 (24.6%)	10,233 (26.5%)	28,591 (12.6%)	3149 (6.9%)	4013 (8.8%)	5108 (11.3%)	6759 (14.9%)	9562 (21.1%)
CRP, median (IQR)	1.29 (0.66–2.53)	0.87 (0.45–1.80)	1.11 (0.59–2.23)	1.32 (0.70–2.56)	1.48 (0.80–2.80)	1.69 (0.94–3.13)	1.38 (0.65–2.98)	0.75 (0.40–1.53)	1.04 (0.53–2.16)	1.38 (0.68–2.82)	1.82 (0.91–3.66)	2.46 (1.26–4.73)

Hypertension was defined as systolic/diastolic blood pressure ≥ 140/90 mm Hg and/or self-reported use of anti-hypertensive medications. Diabetes was defined as self-reported diabetes and/or HbA1c ≥ 6.5% and/or self-reported use of anti-diabetics. The BMI range was < 24.9, 25.0–29.9, and ≥ 30 kg/m^2^ for underweight/normal, overweight and obese, respectively

AIP: atherogenic index of plasma: BMI: body mass index: CRP: C-reactive protein: CVD: cardiovascular disease: HDL: high-density-lipoprotein cholesterol: IQR: interquartile range: LLT: lipid-lowering treatment: SD: standard deviation

### Association between AIP and newly diagnosed AAA in males and females

During a median follow-up of 15.3 (IQR 14.5–16.0) years, 2355 newly diagnosed AAA cases were recorded, with 1931 (1.00%) new cases in males and 424 (0.19%) in females.

**Fig. 1:**
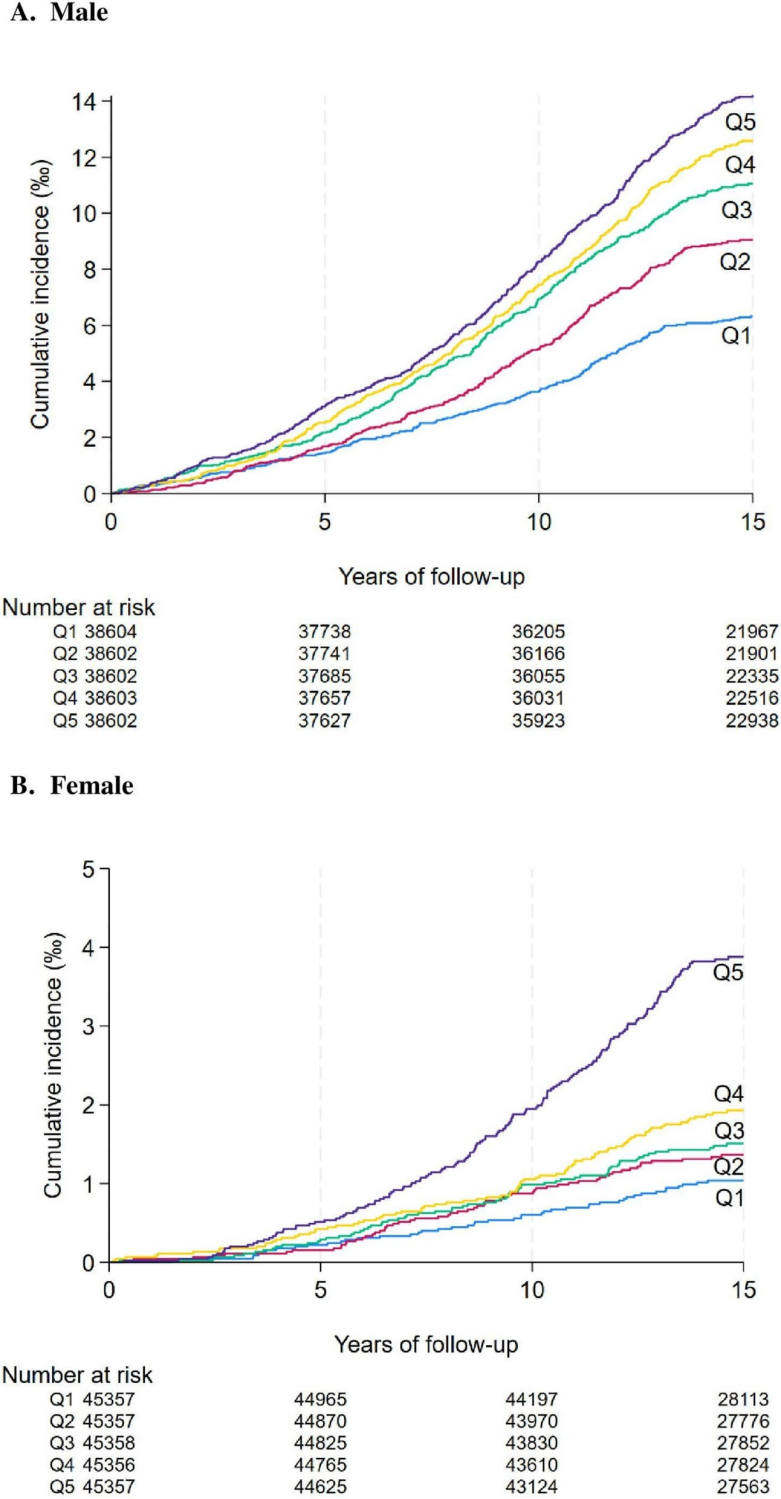
Nelson-Aalen cumulative hazard curves for incident AAA by quintiles of AIP and sex. Nelson-Aalen cumulative hazard curves for incident AAA were presented in (**A**) male (**B**) female, separately. Q1 to Q5 represent the lowest quintile of AIP to the highest quintile of AIP. Abbreviations: AAA, abdominal aortic aneurysm; AIP, atherogenic index of plasma

**Fig. 2:**
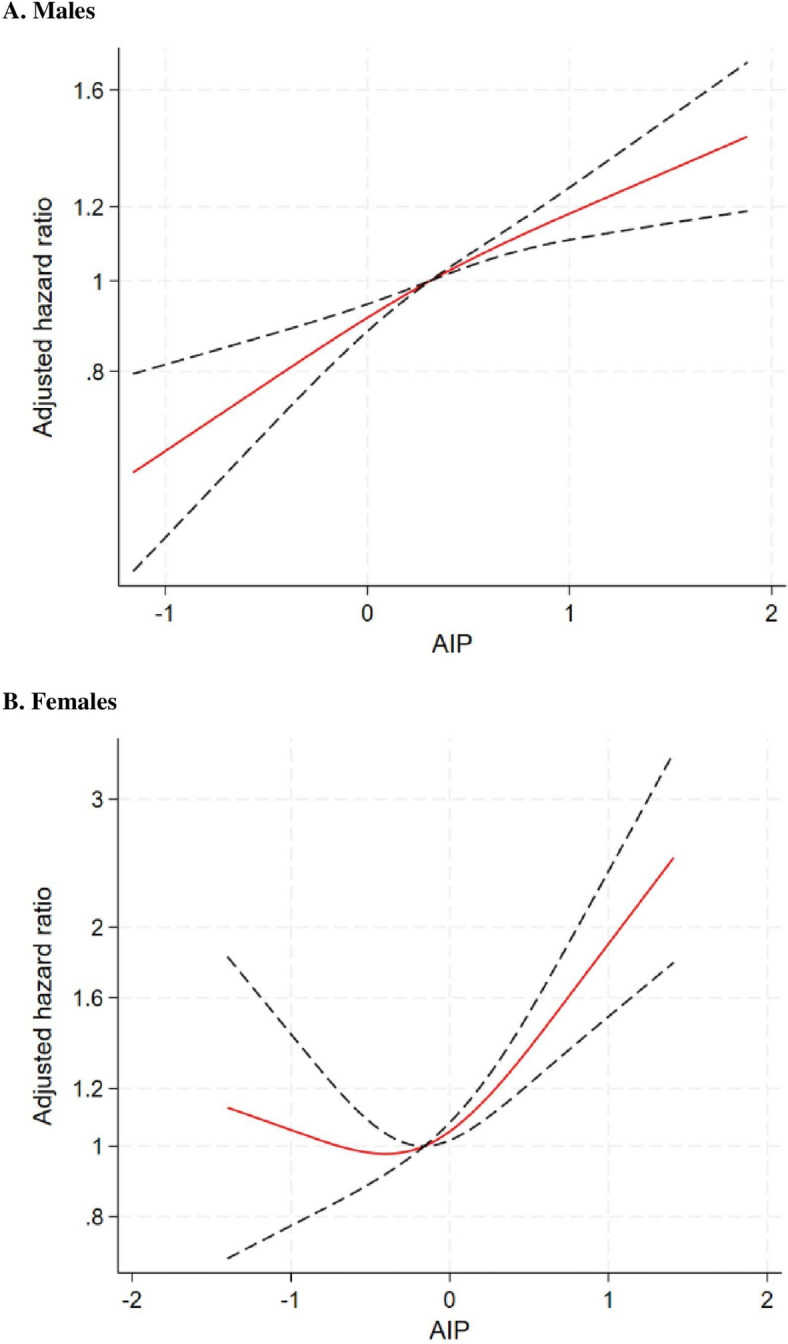
Restricted cubic splines for the association between AIP and new onset AAA in the total population and by sex. The splines were truncated at 1% and 99% to remove the extreme values. Cutoff for each spline was selected as the median value of atherogenic index of plasma (AIP) in the total population and sub-populations (male [fig 2A]: 0.31 female [fig 2B]: -0.16,)

In male participants (Table 2**)**, the incidence rate from the lowest to highest quintile of AIP was 4.1, 5.8, 7.1, 8.1, and 9.1 per 10,000 person-years. In the unadjusted model, per 1-SD increase in AIP was associated with 29% increased risk of AAA (95% CI 1.24–1.35, *p* < 0.001). This association was slightly attenuated in the adjusted models, with HR (95% CI) of 1.22 (1.16–1.28) in the model adjusting for non-lipid covariates and of 1.19 (1.13–1.25) in the model adjusting for both non-lipid covariates and LDL-c, both *p* < 0.001. In the fully adjusted model, compared to the lowest quintile, individuals in the 2nd to 5 th quintiles had HRs of 1.22, 1.37, 1.47, and 1.67, respectively, for developing AAA (*p* for linear trend < 0.001). The Nelson–Aalen cumulative hazard curve revealed a higher cumulative incidence of AAA in participants in the higher AIP quintile (Fig. 1A) Restricted cubic spline revealed almost a linear, monotonic relationship between higher AIP levels and a greater risk of AAA development (Fig. 2A).

**Table 2:** Associations between AIP and newly diagnosed AAA

Quintile of AIP		HR (95% CI)		
	Cases/Incidence rate	Unadjusted model	Fully adjusted model without LDL-c	Fully adjusted model with LDL-c
Male				
Q1 (*n* = 38,604)	231 (4.1)	Ref	Ref	Ref
Q2 (*n* = 38,602)	330 (5.8)	1.43 (1.21–1.69)	1.25 (1.06–1.49)	1.22 (1.03–1.44)
Q3 (*n* = 38,602)	402 (7.1)	1.74 (1.48–2.05)	1.44 (1.22–1.70)	1.37 (1.16–1.61)
Q4 (*n* = 38,603)	455 (8.1)	1.98 (1.69–2.32)	1.56 (1.33–1.84)	1.47 (1.24–1.73)
Q5 (*n* = 38,602)	513 (9.1)	2.23 (1.91–2.61)	1.79 (1.52–2.11)	1.67 (1.41–1.96)
P for linear trend		< 0.001	< 0.001	< 0.001
Continuous (per 1-SD increase), p		1.29 (1.24–1.35) < 0.001	1.22 (1.16–1.28) < 0.001	1.19 (1.13–1.25) < 0.001
Female				
Q1 (*n* = 45,357)	46 (0.7)	Ref	Ref	Ref
Q2 (*n* = 45,357)	61 (0.9)	1.33 (0.91–1.95)	1.05 (0.71–1.54)	1.05 (0.71–1.54)
Q3 (*n* = 45,358)	66 (1.0)	1.45 (0.99–2.11)	0.98 (0.67–1.44)	0.98 (0.67–1.44)
Q4 (*n* = 45,356)	84 (1.2)	1.85 (1.29–2.65)	1.06 (0.73–1.54)	1.06 (0.73–1.55)
Q5 (*n* = 45,357)	167 (2.5)	3.71 (2.68–5.14)	1.76 (1.23–2.50)	1.75 (1.22–2.52)
P for linear trend		< 0.001	< 0.001	< 0.001
Continuous (per 1-SD increase), p		1.60 (1.46–1.75) < 0.001	1.28 (1.15–1.42) < 0.001	1.28 (1.15–1.43) < 0.001

Incidence rate was rate of events per 10,000 person-years. Covariates controlled in the fully adjusted model without LDL-c included baseline age, race, smoking status (never, former, current), alcohol consumption (never, former, current), education level (university or college degree, below university or college degree), Townsend deprivation index (lower than median, median or higher), hypertension, diabetes, cardiovascular disease history, body mass index (BMI) categories (< 25 kg/m^2^ underweight or normal, 25 to < 30 kg/m^2^ overweight, ≥ 30 kg/m^2^ obese), self-reported use of lipid-lowering medication, C-reactive protein levels

AAA: abdominal aortic aneurysm; AIP: atherogenic index of plasma; CI: confidence interval; HR: hazard ratio; LDL-c: low-density lipoprotein cholesterol; SD: standard deviation; Q: Quintile

In female participants (Table 2), the incidence rate from the lowest to highest quintile of AIP was 0.7, 0.9, 1.0, 1.2, and 2.5 per 10,000 person-years. In the unadjusted model, per 1-SD increase in AIP was associated with 60% increased risk of AAA (95% CI 1.46–1.75, *p* < 0.001). This association was similarly weakened in the adjusted models, with HR (95% CI) of equally 1.28, both *p* < 0.001. In the fully adjusted model, compared to the lowest quintile, individuals in the 2nd to 5 th quintiles had HRs of 1.05, 0.98, 1.06, and 1.75, respectively, for developing AAA (*p* for linear trend < 0.001). The Nelson–Aalen cumulative hazard curve revealed that participants in the highest AIP quintile had notably higher incidence of AAA compared with other quintiles. Those in the lowest AIP quintile had the lowest incidence of AAA (Fig. 1B). The restricted cubic spline analysis revealed a J-shaped relationship between AIP levels and AAA risk. AAA risk remained stable at lower AIP levels but increased sharply when AIP exceeded − 0.3 (a value within the third quintile) (Fig. 2B).

### Subgroup analysis

**Fig. 3:**
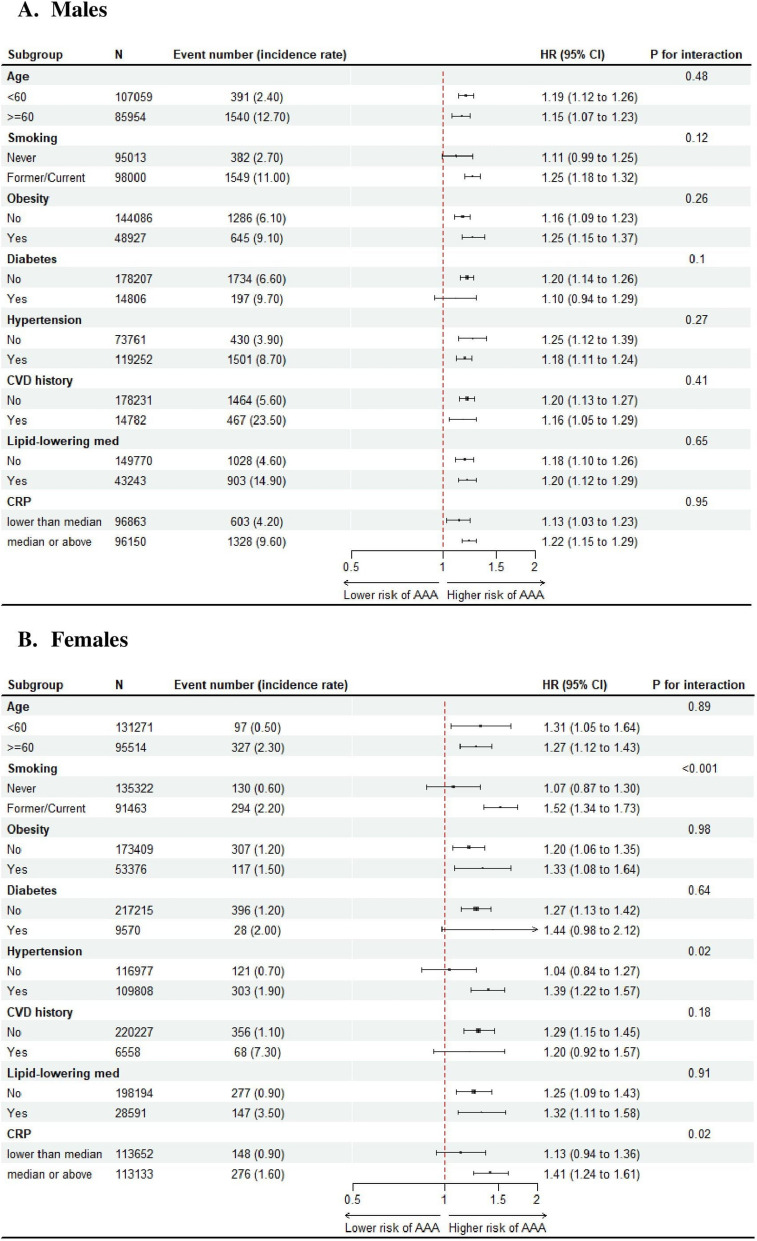
Subgroup analysis in males and females. Results of subgroup analysis by major risk factors were presented in male (**A**) and female (**B**), separately. Abbreviations: CRP, C-reactive protein; CVD, cardiovascular disease

Subgroup analyses found no interaction between AIP and any vascular factors in males (Fig. 3A). However, there was a significant interaction between smoking status and AIP on AAA risk in female participants (p for interaction < 0.001), with the association existing only in individuals who were current or former smoker at baseline (HR per 1 SD increase: 1.52, 95% CI 1.34–1.73) but not in those who had never smoked at baseline (1.07, 0.87 to 1.30) (Fig. 3B).

### Discriminatory ability of AIP

In the model of predicting AAA, typical risk factors including age, smoking and CVD history without AIP already produced a good discriminatory performance in both sexes (Harrell’s C statistic in males and females: 0.808 and 0.811). The performance was further significantly improved by adding AIP in the model in males (change: + 0.005, *p* < 0.001) but not in females (change: + 0.004, *p* = 0.113). However, in female ever-smokers, adding AIP into model including typical risk factors significantly improve model’s discrimination (change: + 0.009, *p* = 0.02), while such improvement was seen in subgroups of female with CVD history and females aged 65 years or order (Supplementary Table 1). In subgroups of older males, male ever-smokers and males with CVD history, adding AIP all significantly improved model performance. The absolute IDI and category-free NRI values were generally consistent with the C-index.

## Discussion

Using data of 419,798 participants enrolled in the UK biobank cohort, this longitudinal population-based study has investigated the sex-stratified association between AIP, a biomarker of atherogenicity, and risk of AAA development, over an average follow-up of 15.3 years. We found a significant dose-dependent association between AIP and AAA risk in male participants and a U-shaped relationship between AIP and AAA risk in female participants. These associations were independent of other vascular risk factors and LDL cholesterol. Male and female participants in the highest quintile of AIP exhibited 67% and 75% increased risk of AAA, respectively, compared to the lowest quintile. Smoking status significantly modified the AIP and AAA association in females, with the association existing only in ever smokers. No other vascular risk factors were identified that had a modifying effect in males and females. AIP significantly improved the discriminatory ability of model comprising age, smoking, and CVD history in males and male subgroups at increased AAA risk, as well as in female ever-smokers.

Our findings suggest that AIP, a marker of plasma atherogenicity, could serve as a valuable biomarker for identifying individuals at higher risk of AAA, particularly in men, who generally have a higher risk than women at the same age and with similar risk factors. The sex-specific differences in the association between AIP and AAA, specifically, the highest quintile of AIP being associated with AAA risk in females and a graded relationship in males, may reflect underlying biological and hormonal differences between the sexes. Women’s generally more favourable lipid profiles and the protective effects of estrogen may contribute to a threshold effect, where only significantly elevated AIP levels are associated with AAA risk. In contrast, men’s higher susceptibility to AAA and differences in risk factor profiles may result in a more continuous relationship between AIP and AAA risk. This hypothesis is further supported by the significant interaction between smoking status and AAA risk in females: increased AAA risk was observed only in current or former smokers, who have a higher baseline AAA risk, but not in never-smokers, who exhibit genuinely low AAA risk. These findings underscore the importance of considering sex as a biological variable in AAA research and risk stratification.

AIP is a surrogate marker of small dense LDL particles, which can easily infiltrate arterial walls and are susceptible to oxidation [15, 16]. Oxidized LDL has been demonstrated that contributes to the formation of foam cells and stimulates immune responses, further leading to the initiation and progression of atherosclerosis [17]. Accumulating evidence has suggested that endothelial dysfunction ubiquitously involves in the pathogenesis of AAA formation, contributing to inflammation and oxidative stress in aortic wall [18]. An animal study found that AIP levels strongly correlate with endothelial dysfunction and aortic degeneration, evidence by the dissociation of elastic fibres and accumulation of collagen in the aortic media. [19]

Atherosclerosis was believed to contribute to AAA, but extant evidence was mixed and inconsistent. Atherosclerosis and AAA shared similar risk factors, such as smoking, older age, and high blood pressure. In biopsy studies, atherosclerosis is often found in aortic wall of AAA patients [2]. In this study, the AIP and AAA association was attenuated but maintained statistical significance after introducing typical vascular risk factors into the model which are highly correlated with atherosclerosis risk. This suggests that AIP-reflected atherosclerosis may contribute to AAA risk, both by interacting with other vascular risk factors and by operating through independent biological mechanisms. However, a recent case–control study comparing AAA patients (case, *n* = 98) to high CVD risk patients (control, *n* = 82) revealed a higher prevalence of carotid atherosclerosis in the control group and similar prevalence in coronary and peripheral atherosclerosis [20]. This study, however, was limited by its small sample size. Another cross-sectional study found a correlation between carotid plaque burden and AAA prevalence but no correlation with aneurismal diameter [21].

Interestingly, we found that AIP adds value to typical AAA risk factors in predicting AAA risk only in female ever-smokers but not in female never-smokers. Smoking is the most dominant risk factor of AAA development, growth and rupture. In a large population-based study, individuals who are current smokers experienced 15- and 7-times higher incidence of AAA compared with never smokers in females and males, respectively [22]. Beyond contributing to atherosclerosis, cigarette toxins have been revealed that can lead to tissue damage the aortic wall by proteolytic enzymes through blocking the active site of α1-antitrypsin [23]. Our findings suggest that AIP may contribute to AAA risk most when it interacts with other ongoing mechanisms involved in the disease's development. Future research is warranted to explore the biological mechanisms behind this and the causal relationship between AIP, other atherosclerosis biomarkers, and AAA. It is also important to determine whether improving AIP levels through pharmacological and non-pharmacological interventions can reduce AAA risk.

To our best knowledge, this is the first-ever epidemiological study investigating the relationship between AIP and development of AAA, providing a valuable mechanical insight into AAA formation and the clinical value of AIP added to other classical risk factors in predicting AAA. While traditional risk factors such as smoking, age, and male sex remain the strongest predictors of AAA, AIP may serve as a complementary biomarker, particularly in women at risk, among whom AAA is less common and potentially underdiagnosed and the necessity of a mass screening is less certain. Future studies should explore whether AIP provides incremental predictive value when added to existing risk scoring systems, including those advanced artificial intelligence (AI)-based models recently developed [24, 25]. If validated, AIP could enhance the identification of asymptomatic individuals who may benefit from targeted ultrasound screening, particularly in intermediate-risk populations. However, further research is needed to determine the optimal integration of AIP into multifactorial risk assessment algorithms and to evaluate its cost-effectiveness in population-based screening programs.

Key strengths of this study include its longitudinal and prospective study design, large sample size, and long-term follow-up. A comprehensive list of potential confounders was meticulously controlled in the Cox regression model to reducing residual confounding bias. Several limitations of this study worth highlighting. This is an observational study thus subjected to inherent residual confounding bias, although we are in attempt to control all other relevant AAA risk factors in the models. No causal relationship can be established. One pre-clinical study using animal models found that AAA had progressed before the focal atherosclerosis became detectable [26]. The AAA diagnosis was based on ICD and operation codes but not ultrasound. While a large-scale ultrasound study would be ideal, administrative data offer a cost-effective alternative and is more realistic and practical. In addition, this study only focused on newly diagnosed AAA cases, whether AIP was associated with AAA growth was not able to be determined.

## Conclusion

This large, prospective cohort study found a significant positive association between AIP and AAA risk in both males and females. Monitoring AIP could help identify high-risk individuals for targeted AAA screening.

## Supplementary Information

Supplementary Material 1.

## Data Availability

No datasets were generated or analysed during the current study.
