# Supplementary material for: Sex-specific association between atherogenic index of plasma and risk of newly diagnosed abdominal aortic aneurysm: a large population-based cohort study
Source: Eur J Med Res. 2025 May 5;30:363. doi: 10.1186/s40001-025-02586-4 (PMC12054325; doi:10.1186/s40001-025-02586-4)
Supplement: Supplementary file 1 — Supplementary Material 1. [file 40001_2025_2586_MOESM1_ESM.docx]

**Supplementary Table 1**. Discriminatory ability of AAA prediction model in males and females.

|  | **Harrell’s C statistic (95% CI)** | **Change in Harrell’s C-statistic, p value** | **Category-free NRI** | **Absolute IDI** |
| --- | --- | --- | --- | --- |
| **Male overall** |  |  |  |  |
| Model including age, smoking, and CVD history | 0.808 (0.799 to 0.817) |  |  |  |
| Model including age, smoking, and CVD history plus AIP | 0.813 (0.804 to 0.822) | + 0.005, p<0.001 | 0.196, p<0.001 | 0.001, p<0.001 |
| **Male aged 65 years or over** |  |  |  |  |
| Model including age, smoking, and CVD history | 0.712 (0.695 to 0.728) |  |  |  |
| Model including age, smoking, and CVD history plus AIP | 0.724 (0.708 to 0.740) | +0.012, p<0.001 | 0.217, p<0.001 | 0.002, p<0.001 |
| **Male ever-smokers** |  |  |  |  |
| Model including age, smoking, and CVD history | 0.773 (0.762 to 0.783) |  |  |  |
| Model including age, smoking, and CVD history plus AIP | 0.782 (0.771 to 0.792) | +0.009, p<0.001 | 0.204, p<0.001 | 0.002, p<0.001 |
| **Male with CVD history** |  |  |  |  |
| Model including age, smoking, and CVD history | 0.709 (0.687 to 0.731) |  |  |  |
| Model including age, smoking, and CVD history plus AIP | 0.715 (0.693 to 0.737) | +0.006, p=0.035 | 0.117, p=0.013 | 0.001, p=0.002 |
| **Female overall** |  |  |  |  |
| Model including age, smoking, and CVD history | 0.811 (0.790 to 0.832) |  |  |  |
| Model including age, smoking, and CVD history plus AIP | 0.815 (0.794 to 0.836) | +0.004, p=0.113 | 0.298, p<0.001 | 0.001, p <0.001 |
| **Female aged 65 years or over** |  |  |  |  |
| Model including age, smoking, and CVD history | 0.751 (0.711 to 0.790) |  |  |  |
| Model including age, smoking, and CVD history plus AIP | 0.758 (0.719 to 0.796) | +0.007, p=0.138 | 0.249, p<0.001 | 0.001, p=0.02 |
| **Female ever-smokers** |  |  |  |  |
| Model including age, smoking, and CVD history | 0.821 (0.797 to 0.845) |  |  |  |
| Model including age, smoking, and CVD history plus AIP | 0.830 (0.807 to 0.853) | +0.009, p=0.020 | 0.395, p<0.001 | 0.001, p=0.003 |
| **Female with CVD history** |  |  |  |  |
| Model including age, smoking, and CVD history | 0.753 (0.685 to 0.820) |  |  |  |
| Model including age, smoking, and CVD history plus AIP | 0.752 (0.684 to 0.820) | -0.0003, p=0.90 | 0.161, p=0.19 | 0.0004, p =0.26 |

Abbreviations: AAA, abdominal aortic aneurysm; AIP, atherogenic index of plasma, CVD, cardiovascular disease.
